# Supplementary material for: The Systems Biology Research Tool: evolvable open-source software
Source: BMC Syst Biol. 2008 Jun 29;2:55. doi: 10.1186/1752-0509-2-55 (PMC2446383; doi:10.1186/1752-0509-2-55)
Supplement: Additional file 1 — SBRT Archive. An archive of the current version of the Systems Biology Research Tool. [file 1752-0509-2-55-S1.zip › sbrt-1.4.0/doc/developers_guide/api/sbrt/kernel/math/stat/proc/MwUAnalysis.html]

MwUAnalysis


|  |  |  |
| --- | --- | --- |
| **Overview** \| **Package** \| **Class** \| **Use** \| **Tree** \| **Deprecated** \| **Index** \| **Help** | |  |
| **PREV CLASS**   NEXT CLASS | **FRAMES**    **NO FRAMES**     **All Classes** |  |
| SUMMARY: NESTED \| FIELD \| CONSTR \| METHOD | DETAIL: FIELD \| CONSTR \| METHOD |  |


---


## sbrt.kernel.math.stat.proc Class MwUAnalysis<V>

```
java.lang.Object
  sbrt.kernel.math.stat.proc.MwUAnalysis<V>
```

**Type Parameters:**: `V` - the variable type.

**All Implemented Interfaces:**: KernelProcess<MannWhitneyResults>

---

``` public class MwUAnalysis<V> extends java.lang.Object implements KernelProcess<MannWhitneyResults> ```

This class is used to perform Mann-Whitney U tests.

**Author:**
:   This class was written and documented by Jeremiah Wright
    while in the Wagner lab.

---

**Constructor Summary**

`MwUAnalysis()`
  
          Constructs a new Mann-Whitney U analysis.


| **Method Summary** | |
| --- | --- |
| `void` | `run(ResultsManager<MannWhitneyResults> resultsManager)`             Computes the Mann-Whitney U statistic of the samples A and B, and adds the results to the provided results manager. |
| `void` | `setSampleA(java.util.List<? extends java.lang.Number> sampleA)`             Sets sample A to the provided list of numbers. |
| `void` | `setSampleB(java.util.List<? extends java.lang.Number> sampleB)`             Sets sample B to the provided list of numbers. |

**Methods inherited from class java.lang.Object**

`clone, equals, finalize, getClass, hashCode, notify, notifyAll, toString, wait, wait, wait`

**Constructor Detail**

### MwUAnalysis

```
public MwUAnalysis()
```

:   Constructs a new Mann-Whitney U analysis.


**Method Detail**

### setSampleA

```
public void setSampleA(java.util.List<? extends java.lang.Number> sampleA)
```

:   Sets sample A to the provided list of numbers.

    :   **Parameters:**: `sampleA` - a list of values.

---


### setSampleB

```
public void setSampleB(java.util.List<? extends java.lang.Number> sampleB)
```

:   Sets sample B to the provided list of numbers.

    :   **Parameters:**: `sampleB` - a list of values.

---


### run

```
public void run(ResultsManager<MannWhitneyResults> resultsManager)
```

:   Computes the Mann-Whitney U statistic of the samples A and B,
    and adds the results to the provided results manager.

    :   **Specified by:**: `run` in interface `KernelProcess<MannWhitneyResults>`
    :   **Parameters:**: `resultsManager` - the results manager to which the computed results are to be added.


---


|  |  |  |
| --- | --- | --- |
| **Overview** \| **Package** \| **Class** \| **Use** \| **Tree** \| **Deprecated** \| **Index** \| **Help** | |  |
| **PREV CLASS**   NEXT CLASS | **FRAMES**    **NO FRAMES**     **All Classes** |  |
| SUMMARY: NESTED \| FIELD \| CONSTR \| METHOD | DETAIL: FIELD \| CONSTR \| METHOD |  |


---
